# The distribution and function of GDE2, a regulator of spinal motor neuron survival, are disrupted in Amyotrophic Lateral Sclerosis

**DOI:** 10.1186/s40478-022-01376-x

**Published:** 2022-05-12

**Authors:** Anna Westerhaus, Thea Joseph, Alison J. Meyers, Yura Jang, Chan Hyun Na, Clinton Cave, Shanthini Sockanathan

**Affiliations:** 1 The Solomon Snyder Department of Neuroscience, Johns Hopkins University School of Medicine, PCTB1004, 725 N. Wolfe Street, Baltimore, MD 21205 USA; 2 Neuroscience Program, Middlebury College, 276 Bicentennial Way, Middlebury, VT 05753 USA; 3 Department of Neurology, Institute for Cell Engineering, Johns Hopkins University School of Medicine, MRB 706, 733 N. Broadway, Baltimore, MD 21205 USA

**Keywords:** GDE2, Motor neuron, Neurodegeneration, GPI-anchored proteins, ALS

## Abstract

Amyotrophic lateral sclerosis (ALS) is a fatal neurodegenerative disease that affects the viability of upper and lower motor neurons. Current options for treatment are limited, necessitating deeper understanding of the mechanisms underlying ALS pathogenesis. Glycerophosphodiester phosphodiesterase 2 (GDE2 or GDPD5) is a six-transmembrane protein that acts on the cell surface to cleave the glycosylphosphatidylinositol (GPI)-anchor that tethers some proteins to the membrane. GDE2 is required for the survival of spinal motor neurons but whether GDE2 neuroprotective activity is disrupted in ALS is not known. We utilized a combination of mouse models and patient post-mortem samples to evaluate GDE2 functionality in ALS. Haplogenetic reduction of GDE2 exacerbated motor neuron degeneration and loss in *SOD1*^G93A^ mice but not in control *SOD1*^WT^ transgenic animals, indicating that GDE2 neuroprotective function is diminished in the context of *SOD1*^G93A^. In tissue samples from patients with ALS, total levels of GDE2 protein were equivalent to healthy controls; however, membrane levels of GDE2 were substantially reduced. Indeed, GDE2 was found to aberrantly accumulate in intracellular compartments of ALS motor cortex, consistent with a disruption of GDE2 function at the cell surface. Supporting the impairment of GDE2 activity in ALS, tandem-mass-tag mass spectrometry revealed a pronounced reduction of GPI-anchored proteins released into the CSF of patients with ALS compared with control patients. Taken together, this study provides cellular and biochemical evidence that GDE2 distribution and activity is disrupted in ALS, supporting the notion that the failure of GDE2-dependent neuroprotective pathways contributes to neurodegeneration and motor neuron loss in disease. These observations highlight the dysregulation of GPI-anchored protein pathways as candidate mediators of disease onset and progression and accordingly, provide new insight into the mechanisms underlying ALS pathogenesis.

**Supplementary Information:**

The online version contains supplementary material available at 10.1186/s40478-022-01376-x.

## Background

Amyotrophic lateral sclerosis (ALS) is a progressive neurodegenerative disease that primarily affects upper (corticospinal) and lower (spinal) motor neurons, ultimately resulting in muscle weakness, paralysis and death [1–5]. Onset of symptoms typically arises in mid-life, with survival in most cases limited to 2–4 years. Approximately 10% of patients with ALS show a genetic component that is inherited in an autosomal dominant fashion and are defined as having familial ALS (fALS) [6]. The remaining 90% of cases have no known family history of ALS and are designated as having sporadic disease (sALS) [6]. There is no cure for ALS. Current treatment options are limited, with approved medications Riluzole and Edaravone providing modest benefits in few patients [7, 8]. Accordingly, there is a pressing need to deepen our understanding of the mechanisms underlying ALS pathogenesis to accelerate the development of better treatments. The study of disease-associated mutations, the most common of which occur in *SOD1* (*Superoxide dismutase 1*)*, C9ORF72* (*C9 open reading frame 72*), *FUS (Fused in sarcoma*) and *TARDBP* (*Transactive response DNA binding protein 43 kDa*) indicate that abnormalities in pathways downstream of these mutated proteins are causal for neurodegeneration in ALS [9–13]. However, whether endogenous neuroprotective pathways that normally maintain neuronal viability throughout life are targeted in disease and contribute to disease pathology remains unclear. Addressing this gap in knowledge may help clarify the complexities of ALS pathologies, provide new insight into disease etiology and progression, and aid in the development of more effective treatments.

Recent studies have identified separate roles for Glycerophosphodiester phosphodiesterase 2 (GDE2, also known as GDPD5) in embryonic spinal motor neuron development and for motor neuron survival in the adult organism [14–17]. GDE2 is a six-transmembrane protein that together with its family members GDE3 and GDE6, are the only known enzymes in vertebrates that function on the cell surface to cleave the glycosylphosphatidylinositol (GPI)-anchor that tethers some proteins to the membrane [16, 18–22]. GDE2 is also suggested to metabolize glycerophosphocholine; however, this observation is observed in kidney cells and its relevance to the nervous system is still unclear [16, 23]. GDE2 is expressed in neurons, in subsets of terminally differentiated oligodendrocytes and in the vascular endothelium, and its expression is complementary to GDE3 (or GDPD2), which is expressed in oligodendrocyte precursor cells (OPCs) and astrocytes [15, 17, 24–26]. The expression of GDE6 in the mammalian nervous system has not been reported. During embryonic neurogenesis, GDE2 is required for the production of late-born motor neuron subtypes of the Lateral Motor Column (LMC) that innervate the limb musculature [15]. Mechanistically, GDE2 drives motor neuron differentiation by downregulating Notch signaling through GPI-anchor cleavage and release of the metalloprotease inhibitor RECK (Reversion inducing cysteine rich protein with Kazal motifs) [16]. The release of RECK from the cell surface disinhibits ADAM10 (A disintegrin and metalloprotease 10), which subsequently cleaves and releases the Notch ligand Delta-like 1 from the cell surface [16]. In the adult spinal cord, GDE2 function is essential for motor neuron viability across spinal motor columns [17]. Mice lacking GDE2 (*Gde2*^*−/−*^) exhibit age-progressive neurodegeneration with characteristic increases in vacuolization, cytoskeletal protein accumulation, microgliosis, and lipofuscin deposition followed by astrogliosis and motor neuron loss [17]. These deficits are accompanied by peripheral restructuring and a progressive erosion of sensorimotor function. Timed genetic ablation of GDE2 that preserves its embryonic roles in motor neuron generation, phenocopied the neurodegenerative changes and motor neuron loss observed in aged *Gde2*^*−/−*^ animals, indicating that GDE2’s role in motor neuron survival is distinct from its function in embryonic neurogenesis [17]. Thus, GDE2 encodes a physiological pathway that is required for motor neuron survival in the adult spinal cord.

The neurodegenerative changes observed in *Gde2*^*−/−*^ animals show pronounced overlap with degenerative hallmarks observed in the spinal cords of mouse models of ALS such as the *SOD1*^G93A^ animal, which harbors a transgene of the human *SOD1* gene carrying the *G93A* mutation associated with fALS [27–29]. Although the progression of neurodegeneration and motor neuron loss in *SOD1*^G93A^ transgenic mice is more rapid, *SOD1*^G93A^ animals exhibit shared neurodegenerative changes with *Gde2*^*−/−*^ mice, specifically, increased vacuolization, inclusions of cytoskeletal proteins, and inflammation [28, 29]. Interestingly, the release of members of the heparan sulfate proteoglycan glypican (GPC) family, which are established GPI-anchored substrates of GDE2, is reduced in *SOD1*^G93A^ lumbar spinal cords, suggesting an erosion of GDE2 function in this animal model [16, 17, 21]. Based on these observations, we hypothesize that GDE2 neuroprotective activity towards spinal motor neurons is diminished in the context of disease pathology, and that this impairment could contribute to disease progression in ALS. However, whether GDE2 function is impaired in ALS has not been addressed.

Here, we utilize the *SOD1*^G93A^ mouse model of fALS combined with samples from patients with ALS to investigate GDE2 functionality in ALS. Genetic reduction of GDE2 exacerbated neurodegenerative changes in *SOD1*^G93A^ animals but not *SOD1*^WT^ controls, indicating GDE2 hypofunction in *SOD1*^G93A^ spinal cords. Analysis of GDE2 expression in postmortem motor cortex samples comparing control patients and patients with ALS showed a pronounced reduction of GDE2 in membrane fractions prepared from patients with ALS although total levels of GDE2 were equivalent between the two groups. Strikingly, GDE2 was found to accumulate in intracellular compartments in sectioned samples of motor cortex of patients with ALS suggesting the disruption of GDE2 activity in ALS. Supporting this notion, unbiased proteomic analysis of cerebrospinal fluid (CSF) samples from healthy controls and patients with ALS show a disproportionate reduction of released GPI-anchored proteins in CSF from patients with ALS. Taken together, these observations suggest that GDE2 function is disrupted in ALS, an outcome consistent with the model that the failure of GDE2-regulated neuroprotective pathways contributes to motor neuron degenerative pathologies in ALS.

## Methods

### Animals

Mice were bred and maintained in accordance with approved Johns Hopkins University Institutional Animal Care and Use Committee protocols. *Gde2* heterozygous (*Gde2*^±^) mice were bred, maintained, and genotyped as described previously [15]. *SOD1*^G93A^ (Jackson Laboratory Strain #002,726), and SOD1^WT^ (Jackson Laboratory Strain #002,297) animals were bred, maintained, and genotyped as described previously [17].

### Human samples

*Postmortem tissue:* Frozen postmortem motor cortex samples and paraffin embedded sections of postmortem motor cortex were obtained from the Target ALS Multicenter Postmortem Tissue Core. Sections of postmortem motor cortex were also obtained from the Johns Hopkins Brain Resource Center. All procedures were performed with appropriate Health Insurance Portability and Accountability Act (HIPAA)-approved autopsy consents. The demographics of postmortem samples used in this study are provided in Additional File 1: Supplementary Table 1. *CSF samples:* CSF samples from 11 control individuals and 20 patients with ALS were obtained from the Northeast Amyotrophic Lateral Sclerosis (NEALS) consortium. Nine additional CSF samples from control individuals were provided by the Advancing Research and Treatment for Frontotemporal Lobar Degeneration Research Consortium (ARTFL) at the University of California San Francisco (UCSF). Study participants provided written informed consent, and all procedures were approved by the respective Institutional Review Boards (IRB). The demographics of CSF samples used in this study are provided in Additional File 2: Supplementary Table 2.

### Immunohistochemistry

#### Paraffin sections (mouse)

Immunostaining was performed as previously described [17]. Briefly slides were deparaffinized in Xylenes, rehydrated, and washed with Phosphate Buffered Saline (PBS) containing 0.3% Triton-X-100 (PBST). Microwave antigen retrieval in 10 mM Sodium citrate followed by blocking in 5% Normal Goat Serum was performed.

#### Paraffin sections (human)

Human motor cortex sections were deparaffinized in Xylenes, then rehydrated in 100% ethanol, 95% ethanol, 70% ethanol, and rinsed with water [30]. Permeabilization was performed with 0.3% PBST followed by antigen retrieval in 10 mM Sodium Citrate with 0.5% Tween-20 (pH 6.0) in a 95 °C water bath. Sections were then washed in PBS followed by overnight blocking at 4 °C in 5% Bovine Serum Albumin (BSA). GDE2 primary antibody (cSS1[30], Covance,1:500) was diluted in 1% BSA and incubated overnight at 4 °C. After blocking endogenous peroxidases with 0.3% hydrogen peroxide and washing with PBS, tissue sections were incubated with horseradish peroxidase (HRP)-conjugated donkey anti-chicken secondary antibody (Jackson ImmunoResearch, 1:500) diluted in 1% BSA for 1 h at room temperature (RT). Sections were washed with PBS, and 3,3′-Diaminobenzidine solution (Sigma-Aldrich, D4168-50) was applied to sections for visualization. Sections were then washed and mounted with ProLong Antifade Gold with DAPI (ThermoFisher, P36931).

#### Primary antibodies

Rabbit Anti-Iba1 (Wako 019–19,741, 1:250), Mouse Anti GFAP (BD 556,328, 1:250), Mouse Anti-phospho-Neurofilament H (Calbiochem NE1022, 1:250), Rabbit Anti-Peripherin (Millipore Sigma AB1530, 1:250), and Chicken anti-human GDE2 (cSS1[30], Covance, 1:500).

#### Secondary antibodies

Jackson ImmunoResearch Goat Anti-Mouse Alexa 488 (115–545-166), Goat Anti Rabbit Alexa 594 (111–585-144), and Peroxidase-conjugated Donkey Anti-Chicken IgY (703–035-155). Brightfield and epifluorescence images were collected at 20 × using a Zeiss Axioskop 2 upright microscope or a Keyence BZ-X710 epifluorescence microscope. Confocal imaging was performed with an Olympus FV3000 Laser scanning confocal microscope. Images were analyzed using ImageJ (NIH) and Matlab Image Processing (Mathworks).

### Image quantification

#### *SOD *Pathology* (mouse)*

The soma and vacuole sizes were calculated from brightfield H&E images. Custom Matlab imaging software was used to threshold and binarize the images to quantify the vacuolization and motor neuron number. A minimum of 12 ventral horns from lumbar spinal cord were analyzed per animal. Neuronal soma were distinguished from glia based on their location in the ventral grey matter and minimum size of 60 μm^2^. We analyzed a minimum of 900 neurons per genetic condition. To isolate large diameter vacuoles indicative of degeneration, vacuoles required a minimum size of 75 μm^2^ to be included in the dataset. Vacuole counts were binned in 3.75 μm^2^ intervals to create a frequency histogram. The number of vacuoles in each bin plus all smaller bins was calculated as a percentage of all detected vacuoles and graphed as a cumulative probability distribution. The distributions of neuronal sizes were compared with an independent samples Kruskal–Wallis Test, and vacuole size distributions were compared with the Kolmogorov–Smirnov test. Manual counts of the mean number of cytoskeletal inclusions were compared with a 2-tailed Student’s t test. Alpha level is 0.05. Error bars represent standard error of the mean unless otherwise indicated.

#### GDE2 distribution* (human)*

Cells with accumulations of GDE2 were manually quantified from paraffin sections of control (n = 7) and ALS patient (n = 10) motor cortices. All quantification of human samples was performed blinded. For each sample, 10–12 regions of interest (0.23 or 0.39 mm^2^) per section were chosen at random to be imaged and analyzed. Accumulations were scored as high intensity if they took up at least 25% of the cell body and were above a relative intensity threshold set to control using ImageJ (NIH). Manual counts of the mean number of high-intensity GDE2 accumulations per mm^2^ were analyzed using a 2-tailed Student’s t test. For this analysis, the alpha level is 0.05, and error bars represent standard error of the mean.

### Western blot analysis

#### Lysate preparation

Human motor cortex samples were either sonicated in RIPA lysis buffer containing protease inhibitor cocktail (Sigma, P8340) or partitioned into detergent-poor and detergent-rich fractions with Triton-X-114 (Sigma, X114) as previously described [16]. Briefly, 2% Triton-X-114 was pre-conditioned in 100 mM Tris–HCl, pH 7.4, 150 mM NaCl buffer. The frozen tissues were sonicated in 1% Triton-X-114 containing protease inhibitor cocktail, followed by centrifugation (16,000 × g, 2 × 10 min, 4 °C). Detergent-rich and detergent-poor phases were separated by incubating the lysates at 30 °C for 10 min. After centrifugation (3000 × g, 3 min, RT), the detergent-rich pellet was collected for analysis of membrane-bound proteins. Batches of human samples were processed at the same time to minimize variability between samples, and protein amounts were standardized using a BCA Protein Assay kit (ThermoFisher, 23,225).

#### Immunoblotting

Samples were subject to reducing SDS-PAGE using 7.5 or 10% Criterion TGX Precast Gels (Bio-Rad Laboratories) in tris/glycine buffer, transferred onto polyvinylidene difluoride membranes at 100 V for 70 min, and blocked with 5% milk in tris-buffered saline containing 0.3% Tween-20 (TBST) for 2–3 h at RT. Membranes were then incubated with primary antibodies diluted in 5% milk in TBST overnight at 4 °C. HRP-conjugated secondary antibodies were diluted in 5% milk in TBST and incubated for 1 h at RT. Membranes were developed using the KwikQuant Imager (Kindle Biosciences) after incubation with enhanced chemiluminescence substrate (Kindle Biosciences, R1004). Imaged blots were analyzed using ImageJ software (NIH).

#### Primary antibodies

Chicken anti-human GDE2 (cSS1, [30] Covance, 1:1000), Rabbit anti-Na/K ATPase (Abcam, ab76020, 1:100,000), and Rabbit anti-GAPDH (Cell Signaling, 8884, 1:5000).

Secondary Antibodies: Peroxidase-conjugated Donkey Anti-Chicken IgY (Jackson ImmunoResearch, 703–035-155, 1:10,000) and Anti-Rabbit IgG-HRP (Kindle Biosciences, R1006, 1:10,000).

### CSF sample preparation and trypsin digestion for MS experiments

Four experimental batches of 10 samples were examined, with each batch including a master pool (MP) sample containing an equal volume of all CSF samples for normalization between batches. The CSF samples were mixed with a urea buffer, composed of 10 M urea/20 mM tris (2-Carboxyethyl) phosphine hydrochloride (TCEP)/80 mM chloroacetamide (CAA) in 100 mM triethylammonium bicarbonate (TEAB), at a one-to-one ratio. Samples were incubated for 1 h at RT for reduction and alkylation. Protein digestion was carried out using LysC (lysyl endopeptidase mass spectrometry (MS) grade, Fujifilm Wako Pure Chemical Industries Co., Ltd., Osaka, Japan) at a one-to-fifty (w/w) ratio for 3 h at 37 °C and subsequently with trypsin digestion (sequencing grade modified trypsin, Promega, Fitchburg, WI, USA) at a one-to-fifty (w/w) ratio at 37 °C overnight after diluting the concentration of urea from 5 to 2 M by adding 50 mM TEAB. Peptides were desalted using C_18_ StageTips (3 M Empore™; 3 M, St. Paul, MN, USA) after acidifying with 1% trifluoroacetic acid (TFA) to the final concentration. The eluted solution containing peptides was dried with a Savant SPD121P SpeedVac concentrator (Thermo Fisher Scientific, San Jose CA) and then stored at ‒80 °C before use.

For tandem mass tag (TMT)-based quantitative MS, the digested peptides from CSF samples were labeled with 11-plex TMT reagents following the manufacturer’s instructions (Thermo Fisher Scientific). The MP sample was labeled with 131C, and CSFs from ALS and control individuals were labeled with the rest of the TMT tags. The labeling reaction was performed for 1 h at RT after mixing each peptide sample in 100 mM TEAB with TMT reagent in acetonitrile (ACN, HPLC grade), and then quenched by adding 1/10 volume of 1 M Tris–HCl (pH 8.0). The TMT labeled peptides were pooled, resuspended with 10 mM TEAB, and then subjected to basic pH reversed-phase liquid chromatography (bRPLC) fractionation to generate fractions on an Agilent 1260 offline HPLC system (Agilent Technologies, Santa Clara, CA, USA), which includes a binary pump, variable wavelength detector, an autosampler, and an automatic fraction collector. The pooled samples were reconstituted in solvent A (10 mM TEAB, pH 8.5) and loaded onto Agilent 300 Extend-C_18_ column (5 µm, 4.6 mm × 250 mm, Agilent Technologies). Peptides were resolved using a gradient of solvent B (10 mM TEAB in 90% ACN, pH 8.5) at a flow rate of 0.3 mL/min over 90 min, collecting 96 fractions. Subsequently, the fractions were concatenated into 24 fractions followed by vacuum drying using a SpeedVac (Thermo Fisher Scientific, San Jose, CA, USA). The dried peptides were suspended in 0.5% formic acid (FA), and 30% of each fraction was injected for MS analysis.

### LC–MS/MS analysis

Peptide samples were analyzed on an Orbitrap Fusion Lumos Tribrid mass spectrometer interfaced with an Ultimate 3000 RSLCnano nanoflow liquid chromatography (LC) system (Thermo Fisher Scientific). The dried 24 fractionated peptides were reconstituted in 0.5% FA and then loaded onto a trap column (Acclaim™ PepMap™ 100 LC C_18_, 5 μm, 100 μm × 2 cm, Thermo Fisher Scientific) at a flow rate of 8 μl/min. Peptides were separated on an analytical column (Easy-Spray™ PepMap™ RSLC C_18_, 2 μm, 75 μm × 50 cm, Thermo Fisher Scientific) at a flow rate of 0.3 μl/min using a linear gradient with mobile phases consisted of 0.1% FA in water and in 95% ACN. The total run time was 120 min. The mass spectrometer was operated in a data-dependent acquisition mode. The MS1 (precursor mass) scan range for a full survey scan was acquired from 300 to 1,800 m*/z* (mass-to-charge ratio) in the “top speed” setting with a resolution of 120,000 at an *m/z* of 200. The AGC target for MS1 was set as 1 × 10^6^ and the maximum injection time was 50 ms. The most intense ions with charge states of 2 to 5 were isolated in a 3-s cycle, fragmented using higher-energy collisional dissociation (HCD) fragmentation with 35% normalized collision energy, and detected at a mass resolution of 50,000 at an *m/z* of 200. The AGC target for MS/MS (MS2, fragment mass) was set as 5 × 10^4^ and the ion filling time was 100 ms. The precursor isolation window was set to 1.6 m*/z* with a 0.4 m*/z* offset. The dynamic exclusion was set for 30 s, and singly charged ions were rejected. Internal calibration was carried out using the lock mass option (*m/z* 445.1200025) from ambient air.

### Database searches for peptide and protein identification

The acquired tandem MS data were searched against the human UniProt database (released in May 2018, containing protein entries with common contaminants) using the SEQUEST search algorithm through the Thermo Proteome Discoverer platform (version 2.2.0.388, Thermo Fisher Scientific) for quantitation and identification. During MS/MS preprocessing, the top 10 peaks in each window of 100 m*/z* were selected for database searches. The search parameters included two maximum missed-cleavage sites by trypsin as a proteolytic enzyme. Carbamidomethyl (+ 57.02146 Da) at cysteine and TMT reagents (+ 229.162932 Da) modification at N-terminus of peptide and lysine residues were set as fixed modifications while oxidation (+ 15.99492 Da) of methionine was a variable modification. For MS data, MS1 error tolerance was set to 10 ppm and the MS/MS error tolerance to 0.02 Da. The minimum peptide length was set to 6 amino acids, and proteins identified by one peptide were filtered out. Both peptides and proteins were filtered at a 1% false discovery rate (FDR). The protein quantification was performed with the following parameters and methods. The most confident centroid option was used for the integration mode while the reporter ion tolerance was set to 20 ppm. MS order was set to MS2. The activation type was set to HCD. The quantification value correction was disabled. Both unique and razor peptides were used for peptide quantification. Protein groups were considered for peptide uniqueness. Missing intensity values were replaced with the minimum value. Reporter ion abundance was computed based on the signal-to-noise ratio. Quantification value corrections for isobaric tags were disabled. The co-isolation threshold was set to 50%. The average reporter signal-to-noise threshold was set to 50. Data normalization was disabled. Protein grouping was performed by applying strict parsimony principle as following; 1) all proteins that share the same set or subset of identified peptides were grouped, 2) protein groups that have no unique peptides among the considered peptides were filtered out, 3) Proteome Discoverer iterated through all spectra and selected which peptide-spectrum match (PSM) to use in ambiguous cases to make a protein group with the highest number of unambiguous and unique peptides, and 4) final protein groups were generated. The Proteome Discoverer summed all the reporter ion abundances of PSMs for the corresponding proteins in a TMT run.

### Statistical analyses of the results from discovery proteomics

Statistical analysis was conducted with the Perseus software package (version 1.6.0.7). Protein abundance values across the samples were divided by those of MP included in each batch, followed by dividing values of each sample by their median value. After log_2_-transformation of all the values, values across proteins were z-score-transformed. The fold changes between the comparison groups were calculated by dividing the average abundance values across the samples of one group by the ones of another group. The *P* values between the comparison groups were calculated with the 2-tailed Student’s t test. The *q*-values for the volcano plots were calculated by significance analysis of microarrays (SAM) and a permutation-based FDR estimation [31].

## Results

### ***GDE2 reduction exacerbates neurodegenerative changes in SOD1***^G93A^***mouse models***

To assess GDE2 function in a disease context, we wanted to ascertain whether known degenerative conditions could reduce GDE2’s neuroprotective capacity towards spinal motor neurons. *SOD1*^G93A^ transgenic animals undergo rapid spinal neurodegeneration that includes vacuolization, inclusions of neurofilament proteins, and inflammation, which leads to spinal motor neuron death [28, 29]. Despite a more prolonged progression and later onset of motor neuron death, degeneration in *Gde2*^−/−^ mice has considerable phenotypic overlap with the *SOD1*^G93A^ model [17]. To test whether the *SOD1*^G93A^ disease state hinders GDE2 function, we compared *Gde2*^+/+^;*SOD1*^G93A^ versus *Gde2*^+/−^;*SOD1*^G93A^ animals. *Gde2* heterozygotes do not exhibit spinal pathology [17]; accordingly, any worsening of pathology in the *Gde2*^+/−^;*SOD1*^G93A^ would suggest that *SOD1*^G93A^ disease conditions diminish GDE2 function below the threshold for haplosufficiency.

**Fig. 1:**
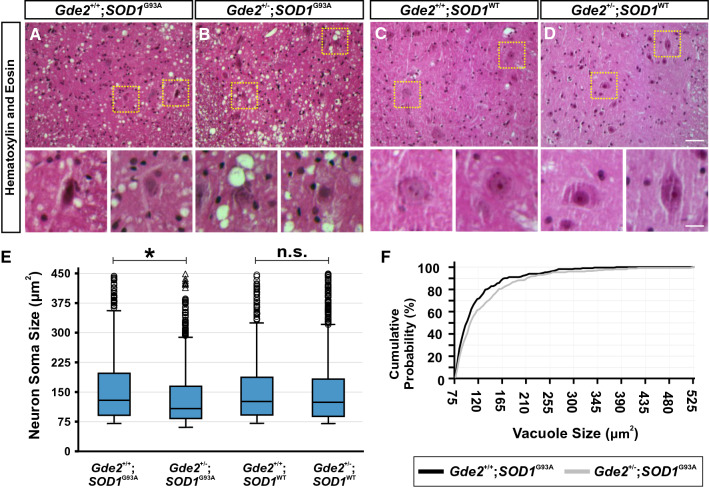
GDE2 is haploinsufficient in *SOD1*^G93A^ transgenic animals. **A-D** Transverse sections of 14 week lumbar spinal cord ventral horns stained with H&E. Yellow boxes are magnified in lower panels, highlighting vacuolated and shrunken neuronal morphology in *Gde2*^+/−^;*SOD1*^G93A^ mice. No morphological changes are seen in *Gde2*^+/−^;*SOD1*^WT^ ventral horn neurons. Scale bar = 50 μm (**A-D**) or 15 μm (insets). **E** Box and whisker plot of neuronal soma sizes. Open circles are large diameter cells above the 95^th^ percentile gates. Open triangles are > 3 times larger than the 75^th^ percentile. *Gde2*^+/−^;*SOD1*^G93A^ neurons are reduced in size compared to *Gde2*^+/+^;*SOD1*^G93A^ controls, Kruskal–Wallis test (*p < 0.01). *Gde2*^+/−^;*SOD1*^WT^ neurons are indistinguishable from *Gde2*^+/+^;*SOD1*^WT^ controls, Kruskal–Wallis test (p = 0.588). No changes are detected in soma size between *Gde2*^+*/*+^*;SOD1*^G93A^ and *Gde2*^+*/*+^*;SOD1*^WT^ as expected at this timepoint [45]. **F** Cumulative probability distribution of vacuole sizes. *Gde2*^+/−^;*SOD1*^G93A^ spinal cords contain larger vacuoles, Kolmogorov-Smirnoff test (p = 0.04). n = 4 *Gde2*^+/+^;*SOD1*^G93A^, 3 *Gde2*^+/−^;*SOD1*^G93A^, 3 *Gde2*^+/+^;*SOD1*^WT^, 4 *Gde2*^+/−^;*SOD1*^WT^

At 14 weeks of age, *Gde2*^+/−^;*SOD1*^G93A^ animals present markedly worsened neuropathology compared to *Gde2*^+/+^;*SOD1*^G93A^ controls (Fig. 1). As neurons degenerate, they shrink in size, succumbing to external phagocytosis and internal apoptotic pathways that produce a pyknotic morphology [32]. *Gde2*^+/−^;*SOD1*^G93A^ mice have significantly reduced neuronal somal size, with a median area of 108 μm^2^ compared to 125 μm^2^ in *Gde2*^+/+^;*SOD1*^G93A^ controls (Fig. 1A, B and E). Importantly, neuronal size is unchanged between *Gde2* wild-types and heterozygotes in the background of the control *SOD1*^WT^ transgene, which does not harbor the disease associated glycine-to-alanine mutation (Fig. 1C-E). Additionally, extracellular vacuolization is more severe in the ventral horn of *Gde2*^+/−^;*SOD1*^G93A^ mice, with an 8% increase in median vacuole diameter (Fig. 1F).

**Fig. 2:**
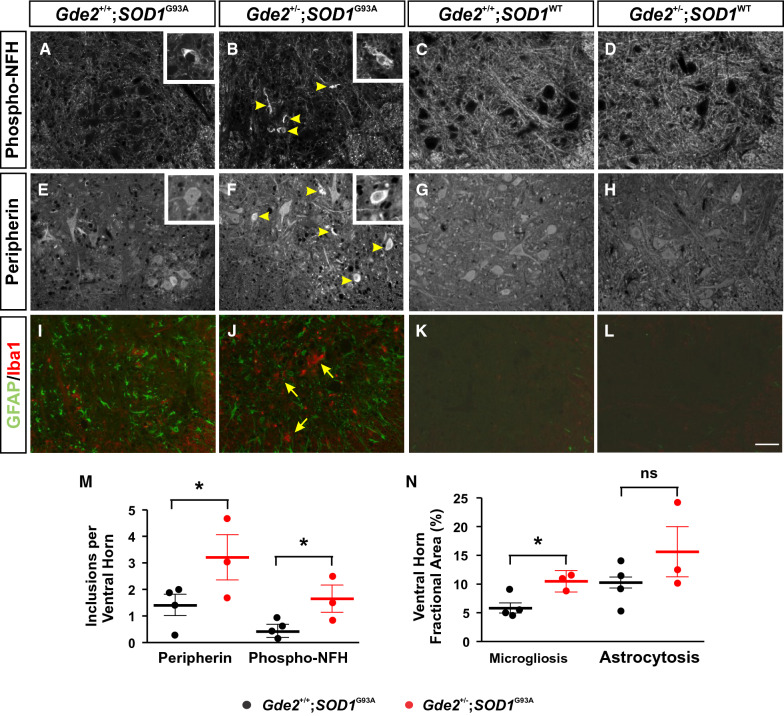
Loss of GDE2 exacerbates *SOD1*^G93A^ pathology. **A-L** Immunostaining of cytoskeletal markers **A-H**; and astrogliosis (GFAP) and microgliosis (Iba1) markers **I-L** in transverse sections of 14-week lumbar spinal cord. Note that images in **K** and **L** were increased in contrast compared with **I** and **J** to show background staining levels in absence of neuroinflammation. Arrowheads in **B** and **F** show increased cytoskeletal inclusions in the *Gde2*^+/−^;*SOD1*^G93A^ spinal cord. Insets shows somal inclusions. Arrows in **J** highlight exacerbated microgliosis. No cytoskeleton accumulations, astrogliosis or microgliosis are evident in the *Gde2*^+/−^;*SOD1*^WT^ and *Gde2*^+/+^;*SOD1*^WT^ controls. **M, N** Graphs show increased peripherin (*p = 0.04), phospho-NFH (*p = 0.03) and microgliosis (*p < 0.01) in *Gde2*^+/−^;*SOD1*^G93A^ spinal cords compared with *Gde2*^+/+^;*SOD1*^G93A^ controls. Astrocyte area fraction is unchanged (p = 0.13). Student’s t test, n = 4 *Gde2*^+/+^;*SOD1*^G93A^, 3 *Gde2*^+/−^;*SOD1*^G93A^, 3 *Gde2*^+/+^;*SOD1*^WT^, 4 *Gde2*^+/−^;*SOD1*^WT^. Scale bar = 50 μm. Graphs represent mean ± SEM

Cytoskeletal accumulations are a prominent hallmark of ALS [33], and spinal motor neuron cell bodies and axons of *SOD1*^G93A^ animals show pronounced inclusions of peripherin and phosphorylated neurofilament-H (phospho-NFH) proteins (Fig. 2A and E). Aged *Gde2*^*−/−*^ animals exhibit similar accumulation of peripherin and phospho-NFH at later stages of adulthood but *Gde2*^+/−^ animals do not [17]. Strikingly, *Gde2*^+/−^;*SOD1*^G93A^ spinal cords show a robust increase in the number of peripherin (3.2 ± 0.8 versus 1.4 ± 0.4) and phospho-NFH (1.6 ± 0.5 versus 0.4 ± 0.2) inclusions in the ventral horn (Fig. 2B, F and M) at 14 weeks of age. In contrast, 14 week *Gde2*^+/+^;*SOD1*^WT^ and *Gde2*^+/−^;*SOD1*^WT^ tissues have no detectable cytoskeletal inclusions (Fig. 2C, D, G and H). The worsening of cellular neurodegenerative phenotypes when GDE2 function is disrupted in *SOD1*^G93A^ animals but not *SOD1*^WT^ mice reinforces the specificity of the effects between *Gde2* and *SOD1*^G93A^.

Reactive gliosis encompasses the morphological thickening, proliferation, and aggregation of astrocytes and microglia upon injury or degeneration, and is indicative of inflammation [34, 35]. We stained sections of spinal cord for Glial Fibrillary Acidic Protein (GFAP) and Ionized calcium-binding adaptor protein-1 (Iba1) to measure astrogliosis and microgliosis, respectively (Fig. 2I and J). Iba1^+^ processes cover 10.4 ± 0.9% of *Gde2*^+/−^;*SOD1*^G93A^ ventral grey matter while accounting for 5.8 ± 0.8% of controls (Fig. 2I, J, and N). GFAP area fraction did not show a significant increase, possibly due to the extreme astrogliosis already present in *Gde2*^+/+^;*SOD1*^G93A^ controls (Fig. 2I, J and N). Evidence of astrogliosis and microgliosis was absent in age-matched *Gde2*^+/+^;*SOD1*^WT^ and *Gde2*^+/−^;*SOD1*^WT^ animals (Fig. 2K and L).

Collectively, these data indicate that GDE2 function is vulnerable to the degenerative stresses in the *SOD1*^G93A^ animal, supporting the possibility that GDE2 hypofunctionality is a component of motor neuron pathologies associated with human neurodegenerative disease.

### GDE2 protein is abnormally distributed in patients with ALS

**Fig. 3:**
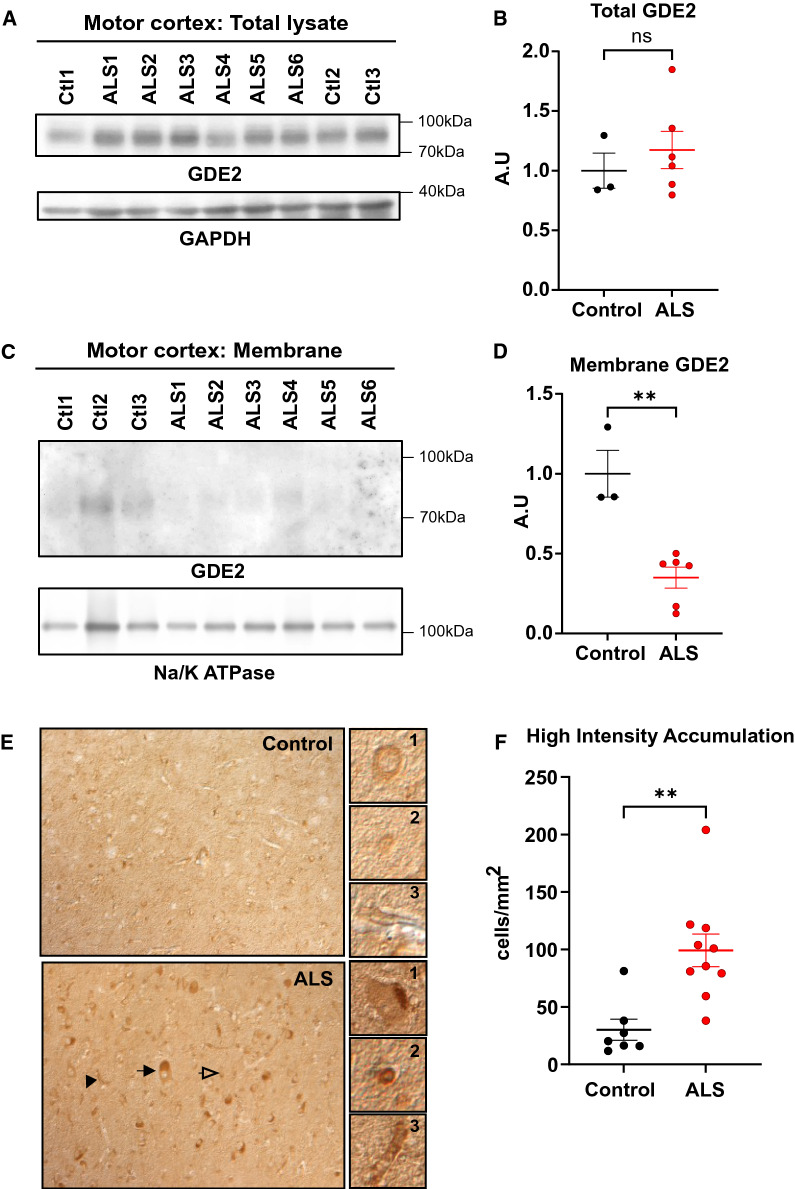
hGDE2 protein distribution is disrupted in ALS. **A** Western blot of protein extracts prepared from postmortem motor cortex of control (Ctl) and patients with ALS. GAPDH serves as a loading control. **B** Quantification of hGDE2 expression relative to GAPDH in total lysate shows no significant (ns) difference in hGDE2 expression between control and ALS patients. Graph represents mean ± SEM. Student’s t test (p = 0.6985), n = 3 Control, 6 ALS. **C** Western blot of Triton-X-114 membrane fractions of motor cortex from control individuals and patients with ALS. Na/K ATPase serves as a loading control and confirms the enrichment of membrane proteins in extracts. **D** Quantification of hGDE2 expression relative to Na/K ATPase in membrane extracts shows reduced hGDE2 expression in membrane fractions from ALS patients compared to control individuals. Graph represents mean ± SEM. Student’s t test (**p = 0.0020), n = 3 Control, 6 ALS. **E** Immunohistochemical staining of postmortem human motor cortex sections of control individuals and patients with ALS. Arrows highlight different types of cells with high-intensity hGDE2 accumulations (black arrow = neuron, clear arrow = glial cell, black arrowhead = blood vessel). Representative images of GDE2 accumulations in these different types of cells compared with control are highlighted in panels 1 (neuron), 2 (glial cell), and 3 (blood vessel). **F** Quantification of the number of high-intensity hGDE2 accumulations in cells from control and ALS sections shows a significant increase of high intensity accumulations of hGDE2 in ALS patients compared to controls. Total number of cells counted: Control = 831 and ALS = 2896. Graph represents mean ± SEM. Student’s t test (**p = 0.0022), n = 7 Control, 10 ALS

Our finding that GDE2 neuroprotective function is impacted in *SOD1*^G93A^ animals motivated us to determine if GDE2 function is disrupted in ALS. We first examined the amounts of GDE2 protein in protein extracts prepared from motor cortex of patients with sporadic ALS and healthy controls (Additional File 1: Supplementary Table 1) using a previously validated antibody that specifically detects human (h) GDE2 (cSS1) [30]. Western blot analysis of protein extracts prepared from the motor cortex of healthy controls and patients with ALS detected equivalent amounts of hGDE2 protein between the two groups (Fig. 3A-B, Additional File 1: Supplementary Table 1). However, Western blot of membrane extracts prepared from postmortem motor cortex samples showed a pronounced reduction of hGDE2 in patients with ALS (Fig. 3C-D, Additional File 1: Supplementary Table 1). These observations suggest that although overall amounts of hGDE2 are unchanged between patient groups, the amounts of functional hGDE2 are reduced in the motor cortex of patients with ALS.

We next used the cSS1 antibody to examine the distribution of hGDE2 in sections of postmortem motor cortex from 7 control patients and 10 patients with ALS. Similar to previous studies, healthy controls showed hGDE2 protein expression in the neuropil and in the cell soma (Fig. 3E), with some cells exhibiting modest accumulation adjacent to the nucleus [30]. However, patients with ALS showed many cells with high levels of hGDE2 accumulation in intracellular compartments (Fig. 3E). These cells include cells with large diameter nuclei that correspond to neurons (Fig. 3E.1), cells with smaller diameter that are consistent with glial cells (Fig. 3E.2), and elongated cells that resemble blood vessels (Fig. 3E.3). These cell types align with the known expression profile of GDE2 in neurons, terminally differentiated oligodendroglia, and vascular endothelia [17, 24, 26]. Quantification of cells with pronounced hGDE2 accumulations showed that there was a robust increase in their numbers in patients with ALS compared with controls (Fig. 3F and Additional File 1: Supplementary Table 1).

These observations reveal that while total levels of hGDE2 are equivalent between controls and patients with ALS, hGDE2 distribution is disrupted with a reduction in membrane-bound hGDE2 and an increase in cytoplasmic hGDE2 accumulations. These abnormalities could imply that hGDE2 function is impaired in ALS.

### Release of GPI-anchored proteins is reduced in CSF of patients with ALS

**Fig. 4:**
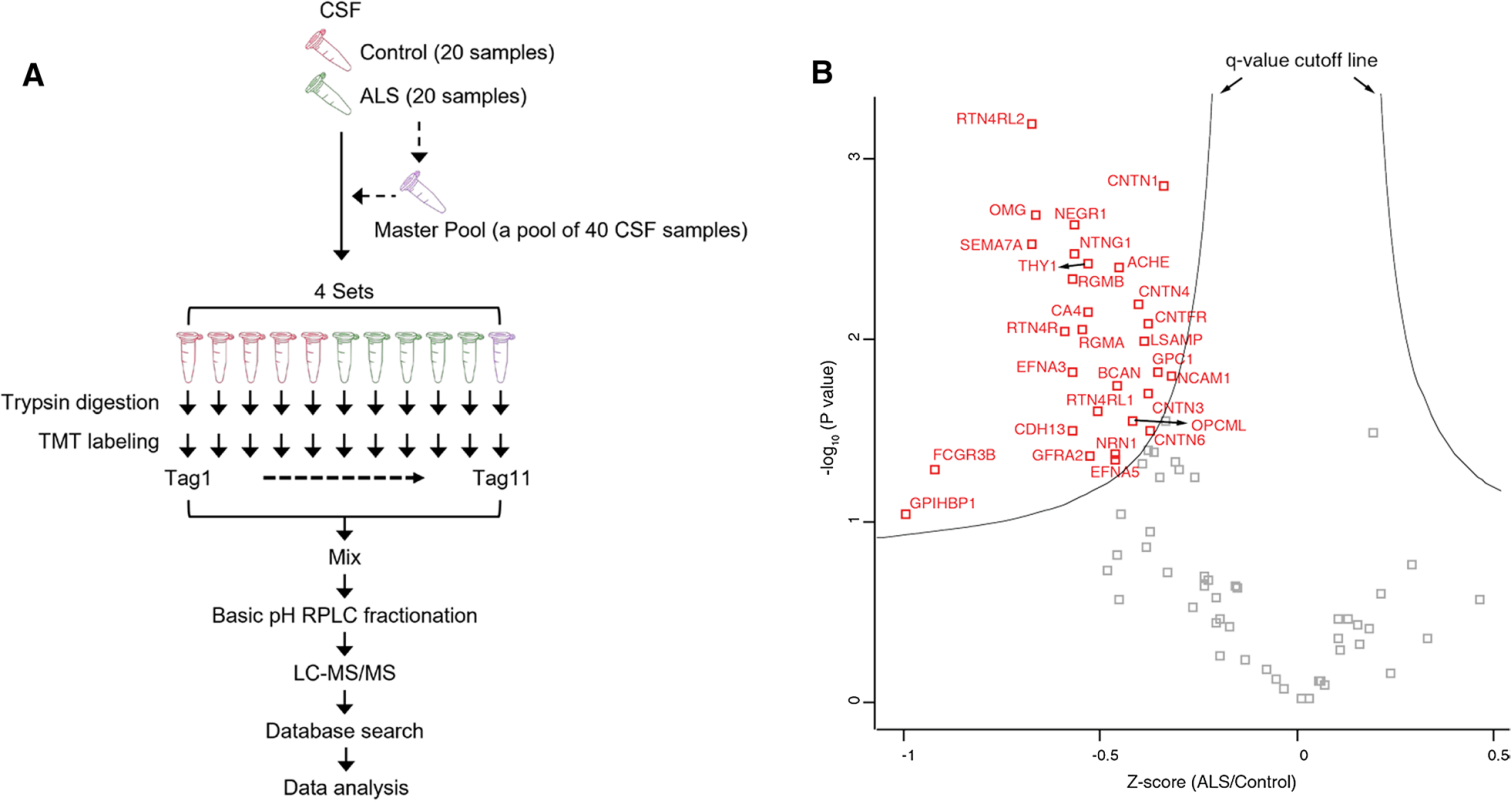
Amounts of GPI-anchored proteins are reduced in CSF of patients with ALS. **A** Schematic outlining the experimental strategy to identify GPI-anchored proteins in patient CSF. Forty CSF samples from 20 control and 20 ALS patients were analyzed for global proteome analysis. Master pool was prepared by pooling equal volumes of CSF from 40 samples, aliquoted into 4 parts and added to one of the 11-plex channels for the normalization between 4 batches of 11-plex TMT experiments. After enzyme digestion of CSF proteins, the resulting peptides were labeled with TMT reagent, followed by pooling the TMT-labeled samples. Subsequently, the pooled peptides were pre-fractionated using basic pH RPLC and each fraction was analyzed by LC–MS/MS. Acquired mass spectra were searched against a reference protein database and statistical analysis was conducted to identify differentially expressed proteins. **B** After normalizing protein abundance values from 40 samples, GPI-anchored proteins were selected, and statistical analysis for the selected proteins was conducted. The proteins outside q-value cutoff lines (q-value 0.1) are differentially expressed proteins

GDE2 acts at the cell surface to cleave the GPI-anchor that tethers some proteins to the plasma membrane and is one of three membrane proteins in vertebrates that regulate surface GPI-anchored protein activity via this mechanism [16, 18, 21]. The reduction in membrane-bound hGDE2 and the corresponding accumulation of hGDE2 in intracellular compartments in patients with ALS suggests that hGDE2 function is disrupted in the context of ALS disease states. If this is the case, then the amount of released GPI-anchored proteins normally cleaved by hGDE2 should be reduced in patients with ALS compared with controls. GPI-anchored proteins that are released from the plasma membrane can partition into the cerebrospinal fluid (CSF). GDE2 is one of two surface GPI-anchor cleaving enzymes expressed in the nervous system; thus, disrupted GDE2 function should result in a reduction of the amount of GPI-anchored proteins in the CSF. To assess global changes in the amounts of GPI-anchored proteins in ALS, we measured the amount of GPI-anchored proteins in patient CSF using tandem-mass-tag (TMT) mass spectrometry (MS), which provides sensitive quantitative measurement of CSF proteins (Fig. 4A). We analyzed a total of 40 CSF samples from 20 control individuals and 20 patients with ALS (Additional File 2: Supplementary Table 2). The 40 samples were split into 4 batches of 10 and were labeled with an 11-plex TMT reagent, which allowed for multiplex running of 11 samples at a time. The master pool (MP), which is a pooled reference sample of equal volumes from all 40 CSF samples, was placed at the 11^th^ channel of each 11-plex TMT experimental set for the purpose of normalization between batches (Fig. 4A). The TMT-labeled samples were pre-fractionated using a basic pH RPLC (reversed-phase liquid chromatography) system for more in-depth protein identification, followed by LC (liquid chromatography)-MS/MS analysis. We identified 3,038 proteins in total, of which 78 were GPI-anchored proteins (Additional File 3: Supplementary Table 3). Twenty-nine GPI-anchored proteins showed a q-value of < 0.1 (Table 1). Notably, amounts of these proteins were found to be decreased in CSF of patients with ALS compared with controls (Fig. 4B, Table 1 and Additional File 3: Supplementary Table 3). Further, searches of expression profiling databases reveal that 27 of the 29 proteins were expressed in cellular sites of GDE2 expression (Table 1). Taken together, these observations reveal a pattern of reduced GPI-anchored protein release in CSF of patients with ALS, correlating with the abnormal intracellular accumulation of GDE2 in ALS patient postmortem samples. These observations provide support that hGDE2 activity and function are disrupted in ALS.

**Table 1:** List of GPI-anchored proteins with q < 0.1 that are differential between CSF from Control individuals and ALS patients. N, neurons; O, oligodendroglial lineage; A, astrocytes; E, endothelial cells; MG, microglia. Cell specific expression profiles were derived from searches of brainrnaseq.org and proteinatlas.org

Protein name	Gene symbol	P value	q-value	Z-score (ALS/control)	Cell Type
Acetylcholinesterase	ACHE	0.0040	0.0535	− 0.451	N, O
Thy-1 membrane glycoprotein (Fragment)	THY1	0.0037	0.0537	− 0.533	N, O, E
Semaphorin-7A	SEMA7A	0.0030	0.0573	− 0.674	N, O, MG
Repulsive guidance molecule A	RGMA	0.0088	0.0576	− 0.547	N, A
Netrin-G1	NTNG1	0.0033	0.0580	− 0.566	N, O
Carbonic anhydrase 4	CA4	0.0069	0.0596	− 0.532	N, MG, E
RGM domain family member B	RGMB	0.0046	0.0597	− 0.569	N, O, A, E
Neuronal growth regulator 1	NEGR1	0.0023	0.0605	− 0.565	N, O, A, E
Ephrin-A3	EFNA3	0.0149	0.0618	− 0.571	N
Reticulon-4 receptor (Fragment)	RTN4R	0.0089	0.0625	− 0.593	N, A
Limbic system-associated membrane protein (Fragment)	LSAMP	0.0103	0.0715	− 0.390	N, O, A
Contactin-1	CNTN1	0.0014	0.0735	− 0.337	N, O, A
Cadherin-13	CDH13	0.0317	0.0736	− 0.569	N, O
Oligodendrocyte-myelin glycoprotein	OMG	0.0021	0.0750	− 0.663	N, O, A
Contactin-4	CNTN4	0.0064	0.0760	− 0.401	N, O
Low affinity immunoglobulin gamma Fc region receptor III-B	FCGR3B	0.0514	0.0771	− 0.922	MG
Ciliary neurotrophic factor receptor subunit alpha	CNTFR	0.0082	0.0773	− 0.377	A
Reticulon-4 receptor-like 1	RTN4RL1	0.0245	0.0816	− 0.509	N
Brevican core protein	BCAN	0.0178	0.0845	− 0.460	A, O
Reticulon-4 receptor-like 2	RTN4RL2	0.0006	0.0860	− 0.677	N, O
Opioid-binding protein/cell adhesion molecule	OPCML	0.0278	0.0877	− 0.419	N, O, E
Glypican-1	GPC1	0.0151	0.0904	− 0.355	N, A
Neuritin	NRN1	0.0419	0.0923	− 0.465	N, O, E
Contactin-3	CNTN3	0.0197	0.0941	− 0.379	N, O
Ephrin-A5	EFNA5	0.0459	0.0967	− 0.463	N, A
GDNF family receptor alpha-2	GFRA2	0.0432	0.0969	− 0.527	N
GPI-anchored high density lipoprotein-binding protein 1	GPIHBP1	0.0905	0.0974	− 0.996	O, E
Contactin-6	CNTN6	0.0317	0.0981	− 0.376	N, O
Neural cell adhesion molecule 1	NCAM1	0.0160	0.0991	− 0.319	N, O, A

## Discussion

Neuronal viability throughout life is maintained through various cellular pathways that mitigate the detrimental consequences of insult and injury. Deeper understanding of how the disruption of these pro-survival pathways contribute to the onset and progression of neurodegenerative disease will provide insight into disease etiology and could inform the design of therapeutic strategies. Previous studies have established that GDE2 is required for the survival of spinal motor neurons but relevance of GDE2 dysfunction to ALS has not been examined [17]. Collectively, using the *SOD1*^G93A^ model of fALS, and patient tissue and CSF samples, we show here that GDE2 distribution and activity is disrupted in ALS. Haplogenetic reduction of GDE2 does not elicit neuropathology or motor neuron loss; however, in *SOD1*^G93A^ animals, genetic reduction of GDE2 resulted in pronounced exacerbation of neurodegenerative phenotypes in line with impaired GDE2 function in the context of disease pathology. Consistent with this logic, GDE2 was found to form intracellular accumulations in postmortem motor cortex of patients with ALS and the amounts of released GPI-anchored proteins in CSF of patients with ALS were reduced. These collective observations provide evidence that GDE2 function is disrupted in ALS, and suggest that GDE2 dysfunction contributes to neuropathologies associated with disease.

Our observation that reducing GDE2 levels in *SOD1*^G93A^ animals aggravates neurodegeneration suggests that GDE2 function is reduced below its threshold of haplosufficiency in disease conditions. Insight into the cellular basis of this dysfunction comes from our analysis of hGDE2 distribution in human postmortem tissues. In contrast to control patients, we find that in motor cortex of patients with ALS, hGDE2 is no longer expressed on the cell surface but instead aberrantly accumulates within cells. Structure–function studies in heterologous cells have shown that surface expression of GDE2 is critical for its GPI-anchor cleavage function [36]. Accordingly, intracellular accumulation of hGDE2 in ALS patient samples is suggestive of a failure of GDE2 enzymatic function in ALS. This notion is borne out by our proteomic studies that show a marked reduction in the amounts of GPI-anchored proteins released into CSF in patients with ALS compared with controls. Proteomic studies of brain extracts reveal that total amounts of GPI-anchored proteins are unchanged between control and ALS patient brain [37]. Specifically, 19 GPI-anchored proteins were identified in human brain, and the total amounts of these proteins were equivalent between control and ALS conditions [37]. In our study, 18 of these proteins were detected in CSF, and 13 were significantly reduced in ALS patient CSF (12 with q < 0.1; 1 with q > 0.1; all p < 0.05). These observations imply that the reduction in the amounts of GPI-anchored proteins in CSF from ALS patients that we observe, is a consequence of reduced release rather than a global loss of protein in disease conditions. GPI-anchored proteins can be released in the CSF by proteolytic cleavage in addition to cleavage at the GPI-anchor. However, because GDE2 and its family member GDE3 are the only known surface GPI-anchor cleaving enzymes in vertebrates that are expressed in the nervous system, we expect that six-transmembrane GDE protein activity would be a major contributor to the amounts of GPI-anchored proteins detected in the CSF. Added support for the concept that disrupted GDE2 enzymatic activity is linked to the intracellular accumulation of GDE2 comes from studies of GDE2 function in Alzheimer’s disease (AD) [30]. GDE2 promotes the non-amyloidogenic processing of the amyloid precursor protein APP by cleavage of the GPI-anchored protein RECK, an inhibitor of the α-secretase ADAM10. In AD, hGDE2 shows pronounced intracellular accumulation in neurons, and this altered distribution is linked to the failed release of RECK and an increase in the amount of membrane RECK in AD patient brain [30]. In contrast, hGDE2 expression is not impaired in postmortem brain of patients with Huntington’s disease (HD) and RECK release is equivalent to control patient samples [30]. These collective observations provide evidence that the disruption of hGDE2 surface expression in disease is synonymous with disrupted hGDE2 enzymatic activity. Combined with our previous observations that GDE2 is required for motor neuron survival [17], our observations support the notion that the failure of hGDE2 activity contributes to neuropathologies and motor neuron loss in ALS. Interestingly, RECK was not identified in the list of GPI-anchored proteins that are differentially expressed in ALS. Because RECK is implicated in Aβ production [30], this would be consistent with the model that GDE2 dysfunction impacts subsets of GPI-anchored proteins in different disease contexts.

Our biochemical analysis of hGDE2 reveals that total amounts of hGDE2 are equivalent between control and ALS postmortem tissues, but that hGDE2 is reduced in the membrane-fraction in the context of ALS. This suggests that hGDE2 is not degraded in disease; instead, rather than being targeted to the membrane, GDE2 may form aggregates within cells, possibly within intracellular compartments. Further studies are needed to determine if hGDE2 has the propensity to aggregate and what factors might drive its intracellular accumulation/aggregation. One potential mechanism that could lead to the intracellular accumulation of GDE2 is the state of cellular redox. GDE2 trafficking to the plasma membrane is normally regulated by the thiol-redox state of two cysteine residues located within its extracellular glycerophosphodiester phosphodiesterase (GDPD) domain [38]. Under oxidative conditions, the ER-resident peroxiredoxin, Prdx4, oxidizes these residues to inhibit GDE2 trafficking to the cell surface, which sequesters GDE2 within the cell [38]. Accordingly, it is plausible that elevated oxidative states observed in disease could block the efficient trafficking of GDE2 to the cell surface, resulting in the intracellular buildup of GDE2 and the erosion of GDE2 function over time.

Studies in human postmortem tissue, mouse models and induced pluripotent stem cells (iPSCs) suggest that in addition to neurons, multiple cell types such as microglia, oligodendrocytes and astrocytes, contribute to neurodegeneration in ALS [39]. For example, mosaic analysis and cell-specific genetic deletions in *SOD1* animal models of fALS suggest that neurons and glia have different contributions to disease onset and progression; however, the mechanisms involved are still unclear [39–44]. Nevertheless, these observations have led to the understanding that ALS is a complex multifactorial disease involving non-cell autonomous mechanisms that ultimately lead to the death of motor neurons and interneurons in the brain and spinal cord. GDE2 is expressed in neurons, terminally differentiated oligodendrocytes and cells of the vascular endothelium [17, 24, 26]. Interestingly, aberrant localization of GDE2 was detected in all of these cell types in the motor cortex of ALS patients. Future studies to examine the cell specific requirements of GDE2 in motor neuron survival will be an important first step in defining the cellular and molecular pathways by which GDE2 regulates neuronal viability.

GPI-anchor cleavage is central to GDE2 function; thus, it is likely that GDE2 regulates the function of specific GPI-anchored protein substrates to mediate neuronal survival. The identities of these proteins are not known but potential candidates that are disease-relevant to ALS would include GPI-anchored proteins that are significantly decreased in the CSF of ALS patients and are expressed in the same cell types as GDE2. The majority of proteins we have identified (27/29) satisfy these criteria (Table 1), including GPC1, a known substrate of GDE2, whose release is also decreased in spinal cord extracts of *SOD1*^G93A^ mice [17]. Notably, 1 of the 29 GPI-anchored proteins identified, CNTFRα, is an astrocyte-specific protein in human brain that is reduced in ALS patient CSF [26]. While GDE2 is not expressed in astrocytes, astrocytes are the main site of GDE3 expression and CNTFRα is a substrate of GDE3 [17, 24, 25]. This raises the possibility that GDE3 function is compromised in ALS, although roles for GDE3 in neurodegeneration have not been established. In addition to providing an entry-point to disease-relevant mechanistic studies of motor neuron survival, our observation that the partitioning of multiple GPI-anchored proteins to the CSF is reduced in ALS indicates potential for their use as disease biomarkers. Future analysis of GPI-anchored protein function and release in ALS could provide new opportunities in clarifying pathogenic mechanisms of ALS and for the development of functional biomarkers of disease.

## Conclusions

Studies in mouse models have established that GDE2 is required for the survival of spinal motor neurons in the adult nervous system. Here, we utilize a combination of ALS mouse models and human samples to examine GDE2 in ALS. Haplogenetic reduction of GDE2 in the *SOD1*^G93A^ mouse model of fALS exacerbates motor neuron degeneration consistent with impaired GDE2 function in pathological contexts. Analysis of hGDE2 protein in human postmortem samples reveals that in ALS, membrane levels of hGDE2 are reduced and that hGDE2 shows abnormal intracellular accumulation. Consistent with disrupted hGDE2 function, the amounts of released GPI-anchored proteins are reduced in CSF of patients with ALS compared with control individuals. These observations suggest that GDE2 function is disrupted in ALS, raising the notion that GDE2’s neuroprotective function is compromised in the context of disease and may contribute to neurodegenerative pathologies in ALS through altered regulation of GPI-anchored protein activity.

## Supplementary Information

**Additional file 1**. Supplementary Table 1: Patient Demographics for postmortem motor cortex samples.

**Additional file 2**. Supplementary Table 2: Patient Demographics for samples used in TMT studies.

**Additional file 3**. Supplementary Table 3: List of differential GPI-anchored proteins detected between ALS and control CSF.

## Data Availability

The datasets supporting the conclusions of this article are included within the article and its additional files.
